# Cadmium-induced apoptosis in neuronal cells is mediated by Fas/FasL-mediated mitochondrial apoptotic signaling pathway

**DOI:** 10.1038/s41598-018-27106-9

**Published:** 2018-06-11

**Authors:** Yan Yuan, Yajing Zhang, Shiwen Zhao, Jie Chen, Jinlong Yang, Tao Wang, Hui Zou, Yi Wang, Jianhong Gu, Xuezhong Liu, Jianchun Bian, Zongping Liu

**Affiliations:** 1 College of Veterinary Medicine, Yangzhou University, Yangzhou, 225009 People’s Republic of China; 2 Jiangsu Co-innovation Center for Prevention and Control of Important Animal Infectious Diseases and Zoonoses, Yangzhou, 225009 People’s Republic of China

## Abstract

Cadmium (Cd) is a toxic metal capable of damaging brain. Studies have demonstrated that Cd can induce apoptosis in neuronal cells. The CD95/APO-1 (Fas)/Fas Ligand (FasL) signaling pathway is one of the primary apoptosis pathways, but the role and regulatory mechanism of this pathway in neuronal cells remain unclear. Here, we demonstrated the underlying mechanism of the Fas/FasL system involving the mitochondrial apoptotic pathway in neuronal cells. Primary rat cerebral cortical neurons and PC12 cells were exposed to Cd, which significantly activated expression of Fas, FasL, Fas-associated death domain (FADD) and cleaved caspase-8. However, expression of cleaved caspase-8 decreased at 20 µM Cd in primary cerebral cortical neurons. Importantly, Cd-induced apoptotic morphological changes and increase in the apoptosis rate were partially blocked by Z-IETD-FMK, which is a specific inhibitor of caspase-8. Cd-mediated increase of apoptosis rate was inhibited by anti-FasL antibody. Furthermore, our data revealed that Z-IETD-FMK also blocked increase of truncated BH3 interacting domain death agonist (tBID)/BID, decrease of the B-cell lymphoma 2 (Bcl-2)/Bcl-2 associate X protein (Bax) ratio and mitochondrial membrane potential (MMP), release of cytochrome c, as well as cleavage of caspase-9/3 and poly (ADP-ribose) polymerase (PARP) induced by Cd. Taken together, our results demonstrate that the Fas/FasL-mediated mitochondrial apoptotic pathway plays an important role in Cd-induced neuronal apoptosis.

## Introduction

Cadmium (Cd) is a toxic heavy metal with common exposure in environmental and industrial pollutant. It can increase the blood brain barrier permeability and lead to neurotoxicity^1,2^. Cd can cause alterations of neurological disorder in animal and humans models, which leads to lower attention, memory deficits, hypernociception and olfactory dysfunction^3,4^. Cd is also a possible etiological factor in neurodegenerative diseases^5^. Thus, it is of great importance to elucidate the underlying mechanisms of Cd-induced neurotoxicity.

Increasing study has shown that Cd can induce neuronal apoptosis^6–9^. However, the precise mechanisms through which Cd induces neuronal apoptosis are unresolved. Apoptosis plays a major regulatory role in homeostasis and development of multicellular organisms^10^. The intrinsic and extrinsic pathways are two well-defined apoptosis regulatory mechanisms. The intrinsic pathway (also called mitochondrial apoptotic pathway) is mediated by the B-cell lymphoma 2(Bcl-2) family proteins. Reduction in the Bcl-2/Bcl-2 associated X protein (Bax) ratio leads to release of cytochrome c from mitochondria into the cytoplasm and activates a caspase cascade that culminates in cellular fragmentation^11^. The extrinsic pathway is initiated by binding of cytokine ligands such as Fas ligand (FasL) and TNF to the death receptors such as CD95/APO-1 (Fas) and TNF receptors. This is followed by caspase-8 activation, which in turn either directly activates caspase-3 or merges with the mitochondrial pathway via cleavage of the Bcl-2 family member, p22 BID^12^. Both pathways converge on activation of caspase-3 which ultimately leads to cell death^13,14^. However, the underlying mechanism of the Fas/FasL apoptotic pathway in neuronal cells remains unclear.

Among cell death receptors, the Fas/FasL system provides an important apoptotic mechanism. Fas is a member of the death receptor family. Fas activation triggers apoptosis via two distinct mechanisms that depend on cell type^15,16^. In type I cells, Fas-induced activation of caspase-8 is sufficient to execute apoptosis by activating effectors caspase-3 and caspase-7. In type II cells, Fas-mediated apoptosis requires involvement of the mitochondrial pathway. The mitochondrial component of the apoptotic process is mediated by truncated BH3 interacting domain death agonist (tBID) translocation from the cytosol to the mitochondria and subsequent cytochrome c release^17^. In our previous report^18,19^, we demonstrated that neuronal apoptosis induced by Cd is associated with activation of the mitochondrial apoptotic pathway by decreasing mitochondrial membrane potential (MMP) and the Bcl-2/Bax ratio, as well as activating caspase-9, caspase-3, and Poly (ADP-ribose) polymerase (PARP) in primary rat cerebral cortical neurons and PC12 cells. However, the underlying mechanism of the Fas/FasL system involving the mitochondrial apoptotic pathway in neuronal cells remains unclear.

The aim of this study was to investigate the role of the Fas/FasL system in Cd-induced neuronal apoptosis and to understand better the relationship between mitochondrial and Fas/FasL apoptotic signaling pathways.

## Results

### Cd induces activation of Fas/FasL signaling

**Figure 1:**
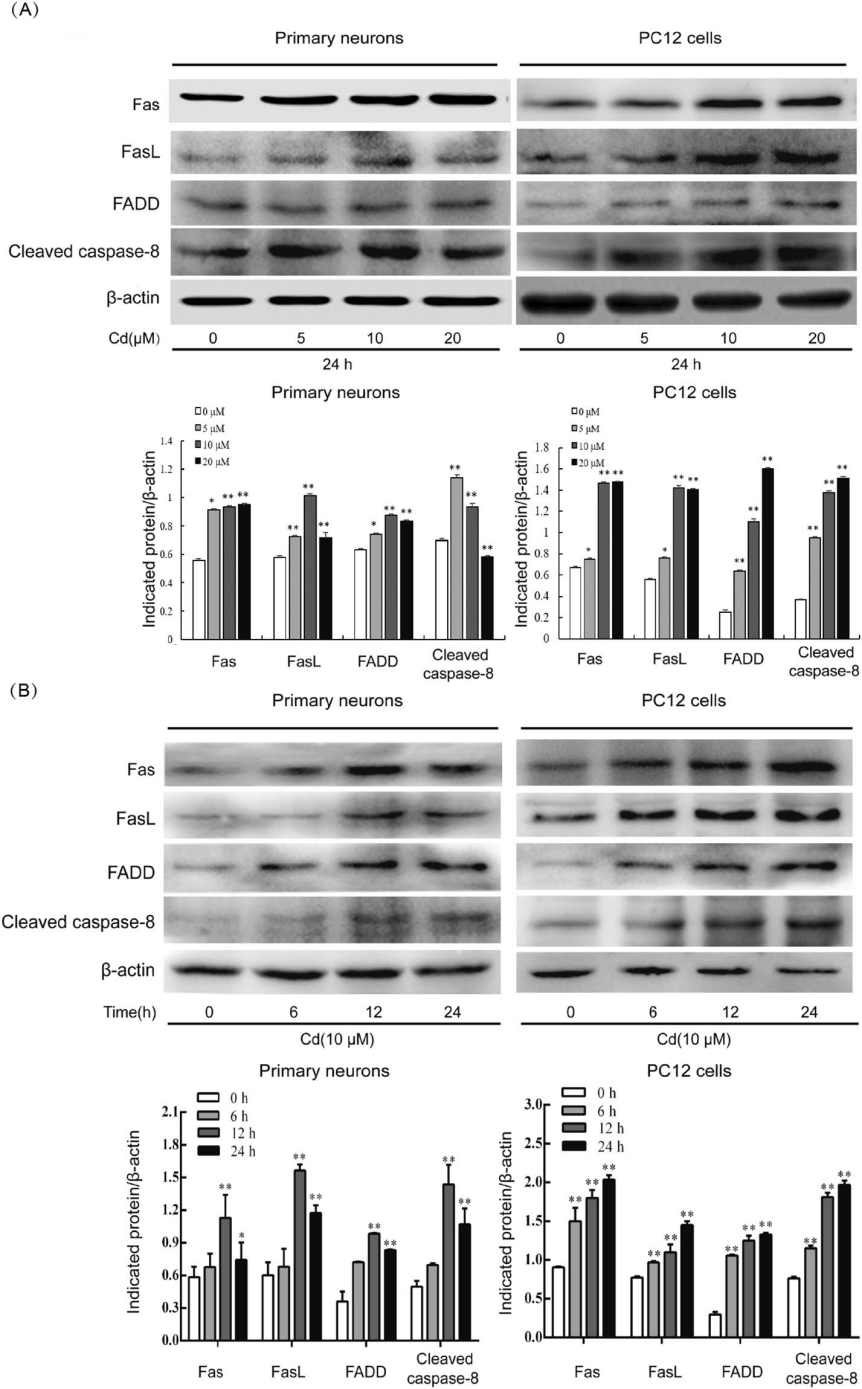
Cd activates the Fas/FasL apoptotic signaling pathway in neuronal cells. Primary rat cerebral cortical neurons and PC12 cells incubated with Cd (0, 5, 10, or 20 µM) for 24 h (**A**), or with Cd (10 µM) for 0, 6, 12, or 24 h (**B**) were harvested, Fas, FasL, FADD, and cleaved caspase-8 protein levels were assessed by western blot analysis using the relevant antibodies. Blots for Fas, FasL, FADD and cleaved caspase-8 were semi-quantified using Image Lab^TM^ software. β-actin was used as internal control. Similar results were observed in at least three independent experiments. Data are expressed as mean ± SD (n = 3) relative to control. *P < 0.05 and **P < 0.01.

Our working hypothesis was that Cd could induce activation of the Fas/FasL apoptotic signaling pathway in neuronal cells. To test this hypothesis, primary neurons and PC12 cells were incubated with Cd (0, 5, 10, or 20 µM) for 24 h or with 10 µM Cd for 0–24 h. Activation of Fas/FasL signaling was detected by western blot. As shown in Fig. 1A, treatment with 5–20 µM Cd for 24 h resulted in an increase in levels of Fas, FasL, and FADD in both primary neurons and PC12 cells. Caspase-8 is a marker for extrinsic apoptosis and its activation was examined. As shown in Fig. 1A, protein levels of cleaved caspase-8 increased after incubation with 5 µM and 10 µM Cd for 24 h in primary neurons, while expression levels decreased at 20 µM Cd compared with the control. In PC12 cells, we observed an increase in expression levels of cleaved caspase-8 at 5–20 µM Cd for 24 h compared with the control. Furthermore, as shown in Fig. 1B, 10 µM Cd induced activation of Fas, FasL, FADD, and caspase-8 within 24 h. These findings clearly indicate that Cd activates Fas/FasL signaling pathways in neuronal cells.

### Cd-induced neuronal cell apoptosis is associated with activation of Fas/FasL signaling

**Figure 2:**
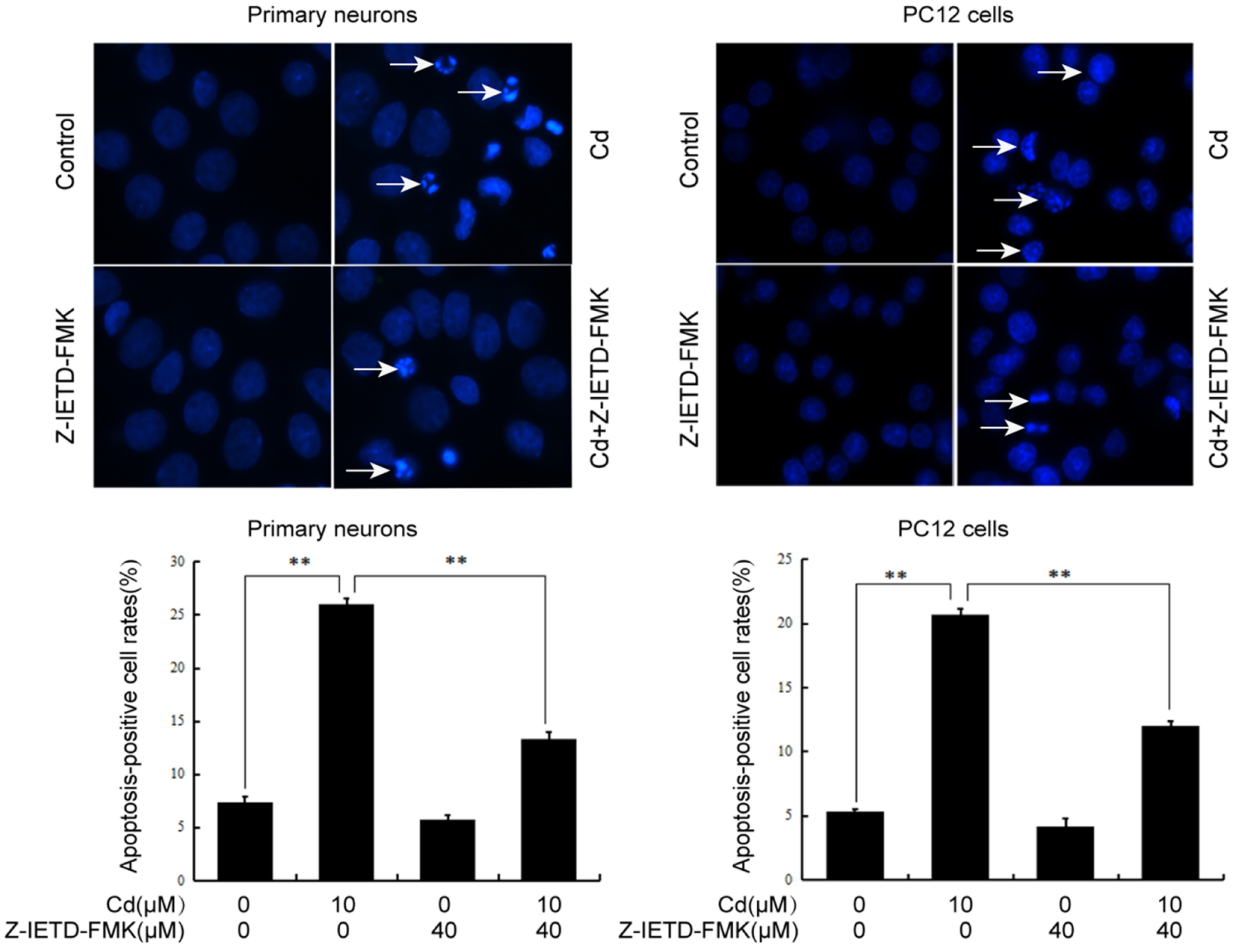
Z-IETD-FMK partially prevents Cd-induced apoptotic morphological changes of neuronal cells. Primary rat cerebral cortical neurons and PC12 cells were pretreated with or without Z-IETD-FMK (40 µM) for 30 min and then exposed to Cd (10 μM) for 24 h. Apoptotic morphological changes of neuronal cells were visualized by fluorescence microscope after Hoechst 33258 staining. Apoptotic cells are indicated by arrows. The original magnification was 200×. Data are expressed as mean ± SD (n = 3). **P < 0.01.

**Figure 3:**
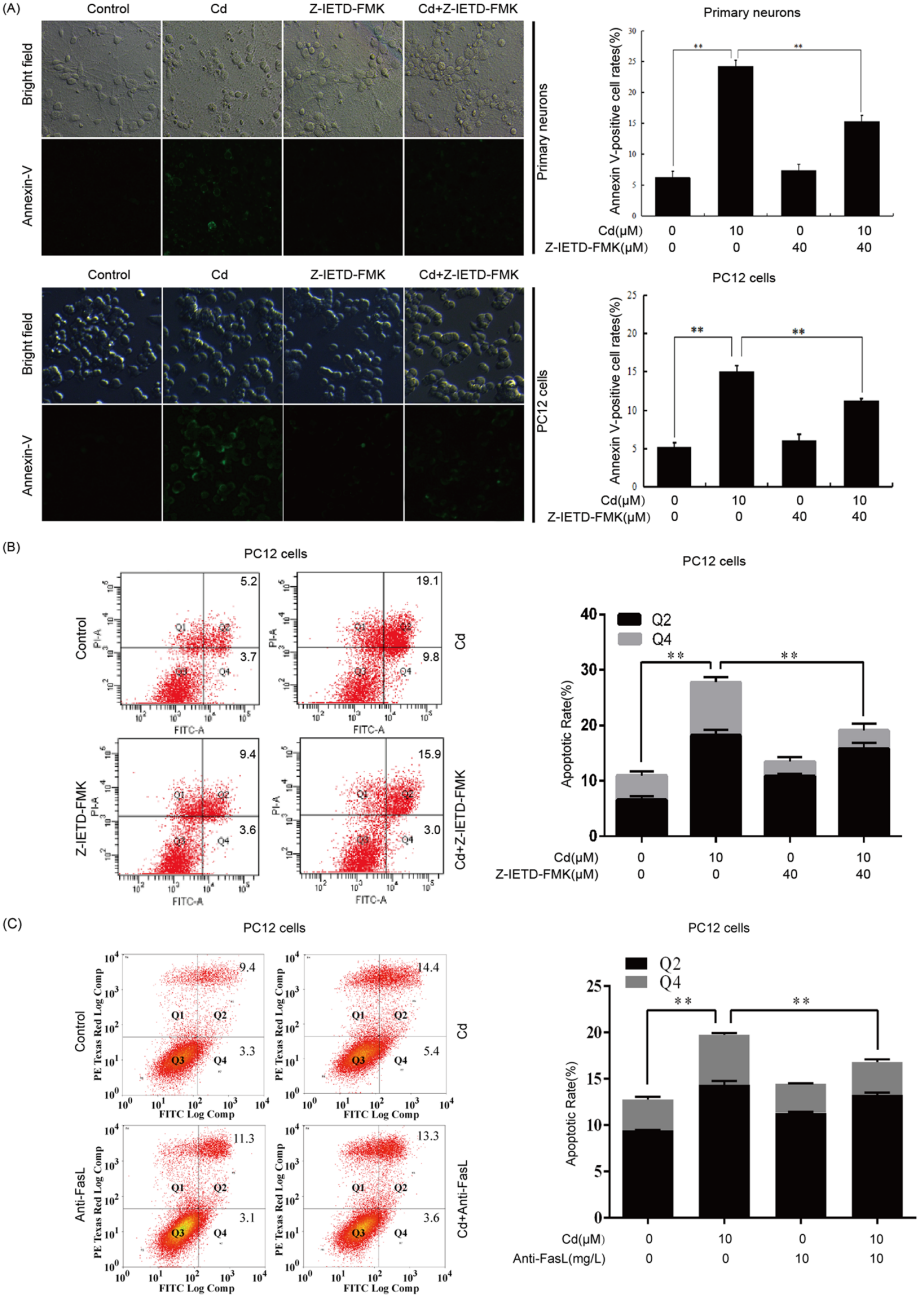
Z-IETD-FMK and anti-FasL antibody partially inhibited Cd-induced apoptosis. (**A**) Primary rat cerebral cortical neurons and PC12 cells were pretreated with Z-IETD-FMK (40 µM) for 30 min prior to treatment with Cd (10 μM) for 24 h. After staining with annexin V-FITC, fluorescence microscopy analysis of neuronal cells was performed with a DMI3000 B inverted phase microscope at 200× magnifications using FITC and bright field filters. PC12 cells were pretreated with (**B**) Z-IETD-FMK (40 µM) or (**C**) anti-FasL (10 mg/L) for 30 min prior to treatment with Cd (10 μM) for 24 h. Following annexin V-FITC and PI double staining, the apoptosis rate of PC12 cells was analyzed by flow cytometry. A representative experimental result is shown. The apoptosis rate was calculated and analyzed using early apoptotic (Q4) and late apoptotic (Q2) cells. Data are expressed as mean ± SD (n = 3). **P < 0.01.

To determine further the role of Fas/FasL signaling in neuronal apoptosis induced by Cd, primary neurons and PC12 cells were pretreated with Z-IETD-FMK (40 µM) for 30 min and then exposed to Cd (10 µM) for 24 h. Apoptotic morphological changes of primary neurons and PC12 cells were detected by Hoechst 33258 staining. In control primary neurons and PC12 cells, nuclear chromatin appeared regular with uniform staining throughout the entire nucleus. In contrast, primary neurons and PC12 cells incubated with 10 µM Cd for 24 h exhibited morphological changes indicative of apoptosis: condensed chromatin appeared at the periphery of the nuclear membrane or appeared as a crescrent shape. Interestingly, the Z-IETD-FMK and Cd co-treated group exhibited fewer changes in nuclei compared with the 10 µM Cd group (Fig. 2). Apoptosis was confirmed by fluorescence microscopy analysis of annexin V-FITC staining and annexin V- FITC/PI assay using flow cytometry analysis. Z-IETD-FMK alone did not appear to alter the number of annexin-V-positive cells or the level of apoptosis. However, Z-IETD-FMK partially prevented the Cd-induced increase in annexin-V-positive cells (Fig. 3A) and the apoptotic rate (Fig. 3B). Furthermore, the increase of apoptotic rate induced by Cd is specifically diminished by anti-FasL antibody in PC12 cells (Fig. 3C). These findings clearly indicate that Cd may at least partially induce apoptosis of the neuronal cells through activation of the Fas/FasL signaling pathway.

### Cd-induced neuronal apoptosis occurs via a Fas/FasL-mediated mitochondrial apoptotic pathway

**Figure 4:**
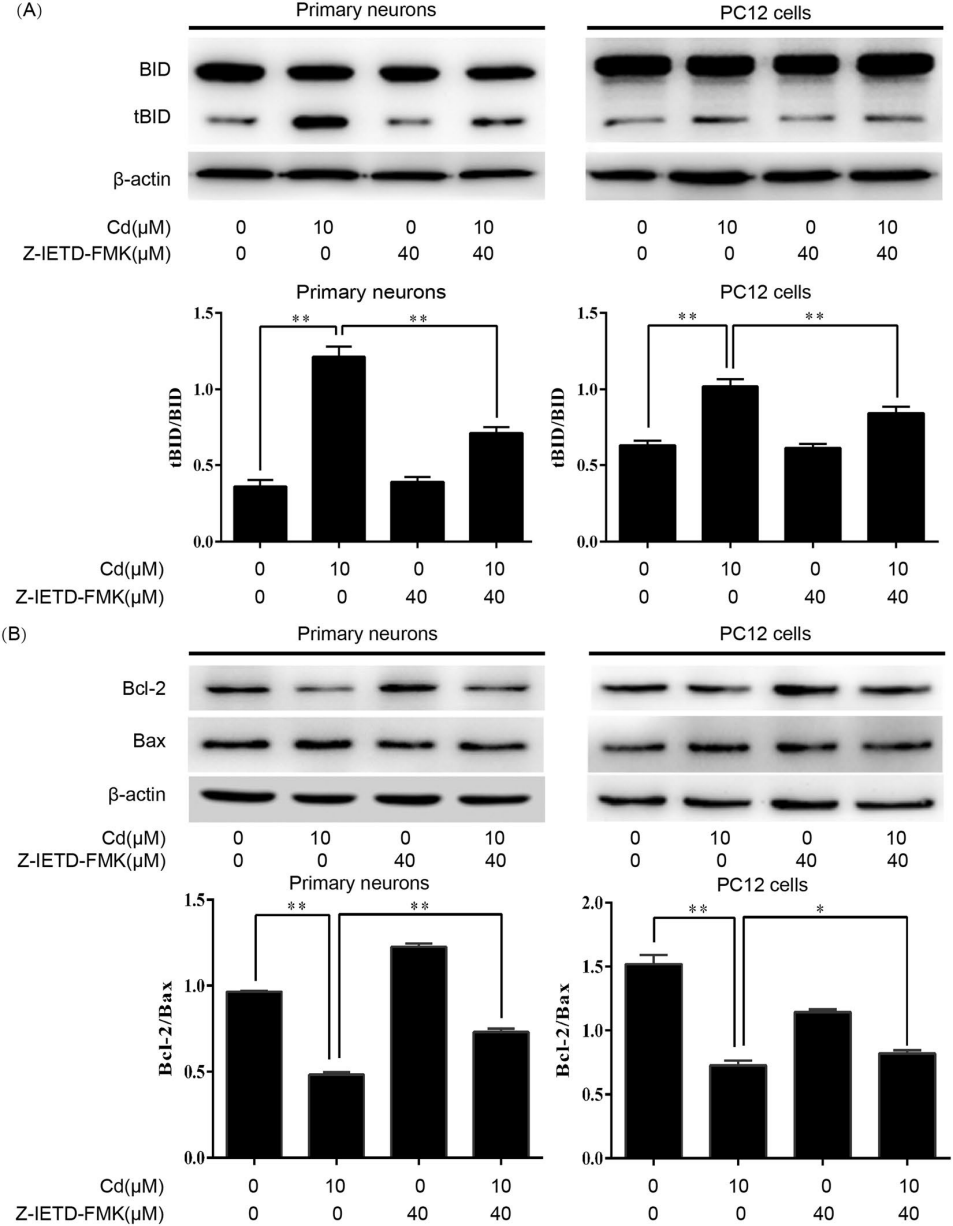
Z-IETD-FMK partially blocks Cd-induced upregulation of tBID/BID, reduction of the Bcl-2/Bax ratio in neuronal cells. Primary rat cerebral cortical neurons and PC12 cells were pretreated with Z-IETD-FMK (40 µM) for 30 min prior to treatment with Cd (10 μM) for 24 h. (**A**) BID and tBID levels and (**B**) Bcl-2 and Bax levels were determined by western blot using the relevant antibodies. Blots for BID and tBID levels (**A**) and Bcl-2 and Bax levels (**B**) in primary neurons and PC12 cells were semi-quantified using Image Lab^TM^ software. β-actin was used as an internal control. Data are expressed as mean ± SD (n = 3). *P < 0.05 and **P < 0.01.

**Figure 5:**
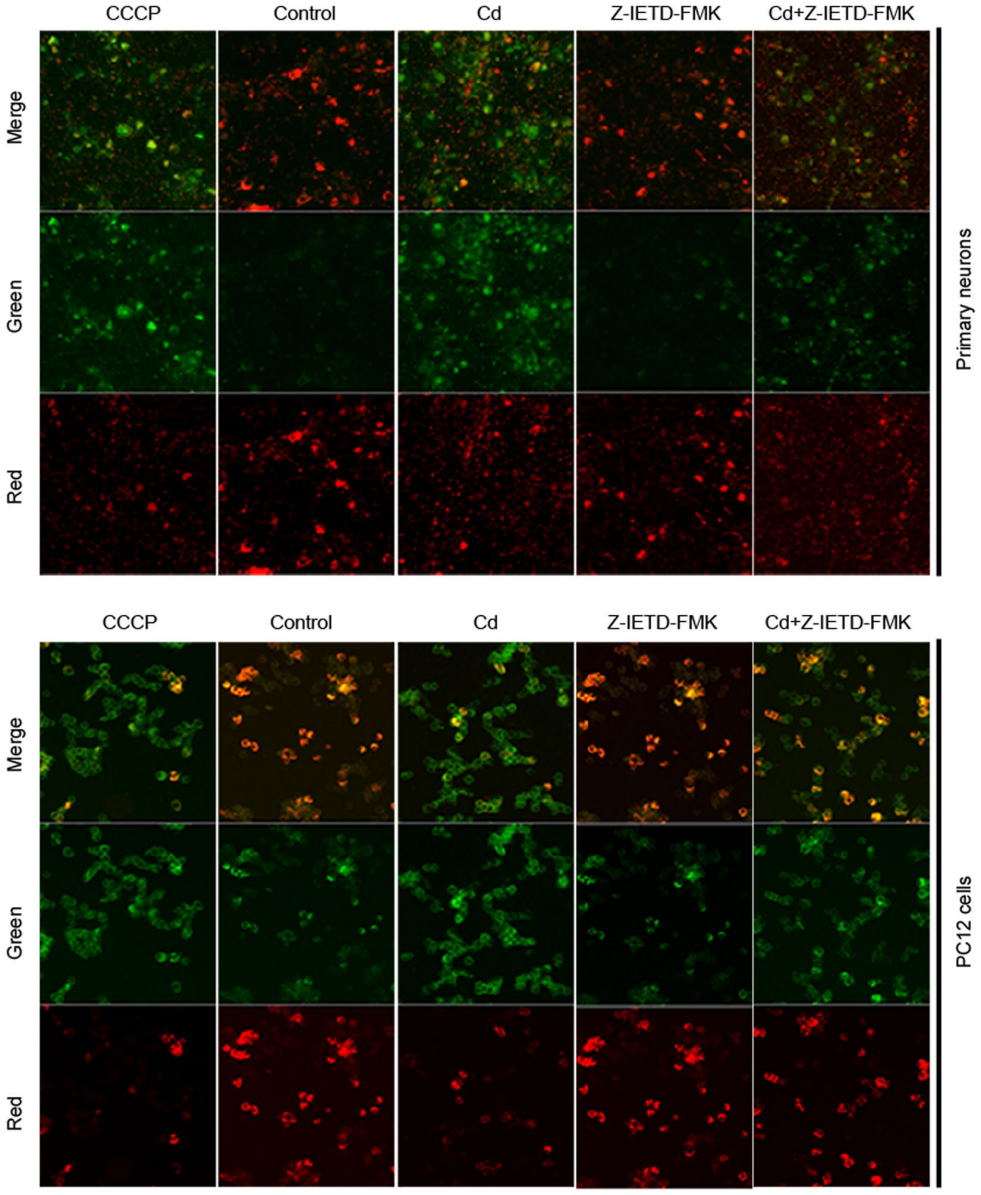
Z-IETK-FMK blocks the decrease in mitochondrial membrane potential (MMP) induced by Cd in neuronal cells. After a 30 min pretreatment with or without Z-IETD-FMK (40 µM), primary rat cerebral cortical neurons and PC12 cells were treated with Cd (10 µM) for 24 h and assayed for MMP by JC-1 staining. Red fluorescence represents the aggregate form (polymer) of JC-1, indicating normal MMP. Green fluorescence represents the monomeric form of JC-1, indicating a decreased MMP. MMP was visualized by a fluorescence microscope and representative images are shown.

**Figure 6:**
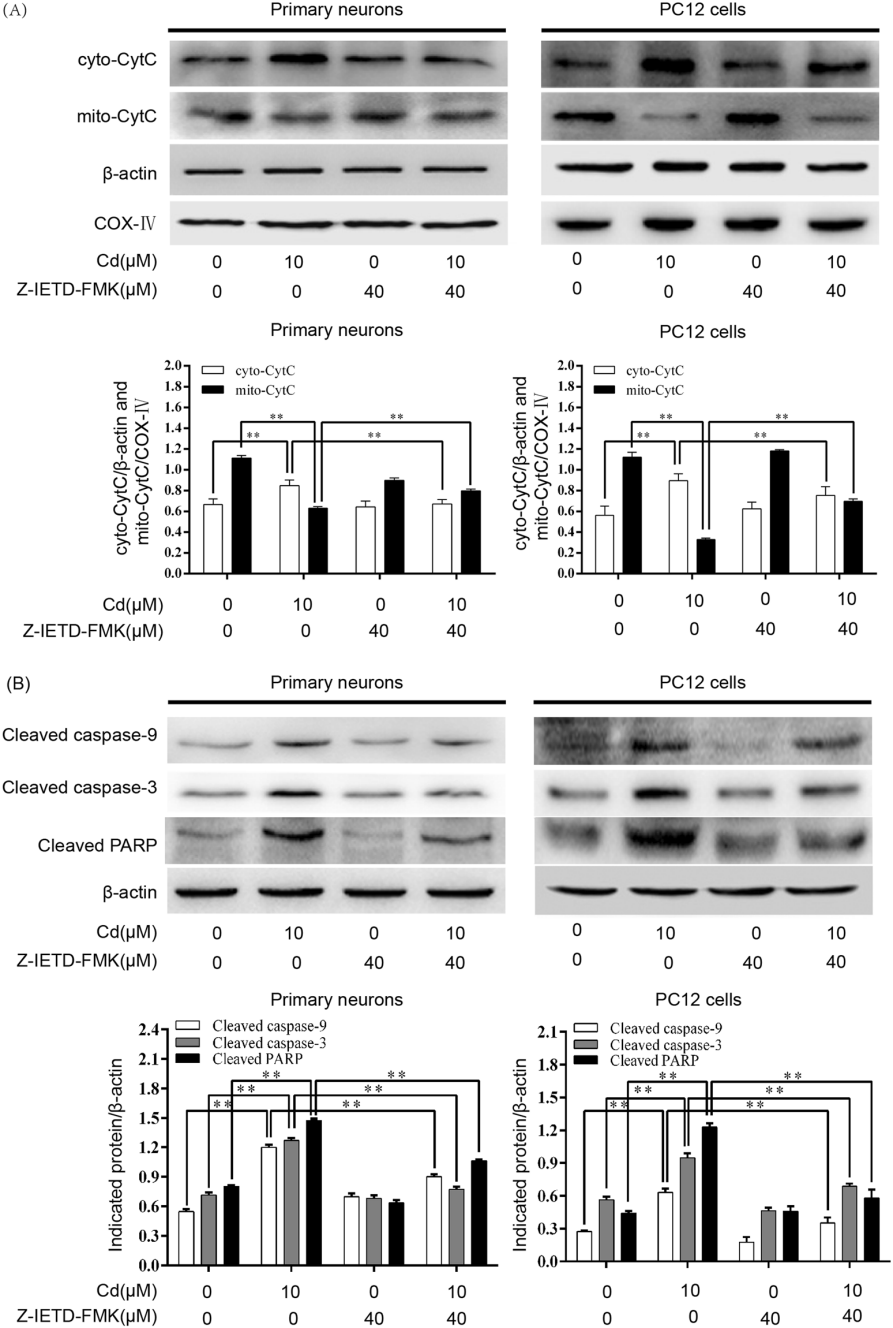
Z-IETD-FMK attenuates Cd-induced cytochrome c release and caspase-9/3, and PARP cleavage in neuronal cells. Primary rat cerebral cortical neurons and PC12 cells were pretreated with Z-IETD-FMK (40 µM) for 30 min prior to treatment with Cd (10 μM) for 24 h. (**A**) The levels of cytosolic cytochrome c (cyto-CytC) and mitochondrial cytochrome c (mito-CytC)) and (**B**) caspase-9, caspase-3, and PARP cleavage were determined by western blot using the relevant antibodies. Cyclooxygenase IV (COX-IV) and β-actin were used as internal controls for the mitochondrial and cytosolic subfractions, respectively. Blots for cytochrome c and caspase-9/3, and PARP cleavage were semi-quantified using Image Lab^TM^ software. Data are expressed as mean ± SD (n = 3). **P < 0.01.

We examined the involvement of Fas/FasL signaling in a Cd-induced mitochondrial apoptotic pathway. As shown in Fig. 4, a Cd-induced increase in the tBID/BID ratio and decrease in the Bcl-2/Bax ratio were significantly attenuated by Z-IETD-FMK. Furthermore, changes in MMP were evaluated by JC-1 staining. An increase in the number of JC-1 monomeric cells (green fluorescence) reflects a decrease in MMP. Compared with control cells, the number of JC-1 monomers was remarkably increased in Cd-treated cells. However, the Cd-induced increase in the monomeric form was attenuated by Z-IETD-FMK (Fig. 5). These results indicate that Z-IETD-FMK significantly blocks the decrease in MMP induced by Cd in neuronal cells. In addition, Cd-induced release of cytochrome c, cleavage of caspase-9/3 and PARP were inhibited by Z-IETD-FMK in neuronal cells, as indicated by western blot (Fig. 6). These findings indicate that neuronal apoptosis induced by Cd via a mitochondrial apoptotic pathway is mediated by Fas/FasL activation.

## Discussion

Cd is a toxic metal capable of entering the brain parenchyma and neurons, which can cause neurological alterations. Growing evidence indicates that Cd severely affects function of the nervous system by inducing neuronal apoptosis^20^. The precise mechanisms involved in Cd-induced apoptosis remain unclear. Apoptosis is a basal process of cell death that occurs through activation of different signaling pathways. The mitochondrial (intrinsic) and death receptor-mediated (extrinsic) pathways are the main apoptosis regulatory mechanisms^21^. In the mitochondria-mediated apoptotic pathway, mitochondria play a key role in apoptosis^22^. Mitochondria are also key intracellular targets of Cd cytotoxicity^23^. Our previous study demonstrated that Cd-induced neuronal (primary rat cerebral cortical neurons and PC12 cells) apoptosis was partially associated with activation of mitochondrial signaling pathways^18,19,24^. Fas is a member of the death receptor family, and the Fas/FasL system is one of the major cell apoptosis pathways. However, the underlying mechanism of the Fas/FasL apoptotic pathway in neuronal cells is unclear. A major finding of this study, which to our knowledge is the first time it has been reported, is that the Fas/FasL mediated mitochondrial apoptotic pathway plays an important role in Cd-induced neuronal apoptosis.

The Fas/FasL system is a key signaling transduction pathway of apoptosis in cells and tissues. The binding of Fas to FasL recruits FADD, and elicits activation of downstream caspase-8, followed by formation of the death-inducing signaling complex (DISC), thus it actives caspase-3, which eventually leads to cell apoptosis^25^. Fas, FasL, FADD, and caspase-8 are effector proteins of the Fas/FasL signaling apoptosis pathway. The Fas/FasL signaling pathway can be activated by Cd^26–28^. In this study, increased neuronal expression of the death receptor Fas, FasL, and downstream effectors, FADD, induced by Cd is the most probable explanation for activation of Fas/FasL signaling pathway (Fig. 1). Caspases are key points of the apoptotic process and play a major role in execution of Fas/FasL-initiated apoptosis. Our results showed that Cd increased protein levels of cleaved caspase-8, which is an important component of the Fas-mediated caspase-dependent apoptosis pathway (Fig. 1). It has been demonstrated that Cd exposure can induce cell apoptosis by activating the Fas/FasL pathway in hepatoma cells^28^. Our previous study showed that Cd induces activation of the Fas/FasL apoptosis pathway in rat proximal tubular cells^29^. However, involvement of the Fas/FasL signaling pathway in neuronal apoptosis induced by Cd is still unclear. In this study, Z-IETD-FMK could prevent Cd-induced apoptotic morphological changes as well as increase in the number of annexin-V-positive cells and the apoptosis rate. For blocking the Fas/FasL interaction, anti-FasL antibody was added to the culture medium before the incubation with Cd. After 24 hours of incubation, the apoptosis rate was analyzed by flow cytometry. The results show that anti-FasL antibody can inhibit Cd-mediated increase of apoptosis rate. These results suggest that Fas/FasL activation might be involved in neuronal apoptosis induced by Cd (Figs 2 and 3). Reactive oxygen species, TNF-α and activated NF-κB are believed to be the main factors responsible for increased expression of FasL and induction of apoptosis^30,31^. Further study is needed for the regulatory mechanism of Fas/FasL signaling pathway induced by Cd in neuronal cells.

Studies have shown that activation of the mitochondrial pathway is required for death receptor-mediated apoptosis in some types of cell (type II cells)^32,33^. In these cells, the link between the death receptor and mitochondrial pathways is caspase-8-mediated cleavage of BID^34^. Increasing evidence has suggested a role of BID in Fas-mediated apoptosis^35,36^. Following death receptor stimulation, cleaved caspase-8 acts on mitochondria through tBID/BID, which bind to proapoptotic protein Bax. This results in a decrease in MMP, release of cytochrome c, and subsequent activation of a downstream caspase, such as caspase-3, which causes apoptosis^37–40^. However, the precise relationship between the two pathways in neuronal cells is not clear. Here, to investigate crosstalk of death receptor signaling with the mitochondrial pathway in neuronal cells, we observed that treatment with Z-IETD-FMK, which is a specific inhibitor of caspase-8, blocked activation of BID and a decrease in the Bcl-2/Bax ratio induced by Cd (Fig. 4). This is consistent with a mechanism in which activation of a death receptor leads to cleavage of procaspase-8, and activated caspase-8 converts BID to tBID. Furthermore, our current data clearly demonstrate that Cd-induced decreases in MMP, release of cytochrome c, and activation of caspase-9/3 and PARP were significantly inhibited by Z-IETD-FMK (Figs 5 and 6), which suggests a link between the death receptor and mitochondrial pathways. These results shown that activation of the extrinsic receptor-mediated pathway leads to apoptosis via Fas/FasL-mediated activation of caspase-8 and BID, which then activates procaspase-9 and procaspase-3 to initiate the caspase cascade required for apoptosis. Taken together, these findings suggest that Cd activates BID, which may direct the Fas/FasL apoptotic signaling toward mitochondrial apoptosis.

In conclusion, we have demonstrated for the first time that Cd promotes apoptosis of neuronal cells by activating the Fas/FasL pathway. Furthermore, the Fas/FasL-mediated mitochondrial apoptotic pathway plays an important role in Cd-induced apoptosis of primary neurons and PC12 cells. Thus, this study provides novel insights into the toxicology of Cd on neuronal cells. Furthermore, the findings suggest that regulation of Cd-activated Fas/FasL signaling may be a potential target in prevention of Cd-induced neurodegenerative diseases.

## Materials and Methods

### Reagents

Cadmium acetate (CdAc_2_), penicillin/streptomycin, Hoechst 33258 stain, poly-D-lysine (PDL) and antibody against Fas-associated death domain (FADD) were purchased from Sigma-Aldrich (St. Louis, MO, USA). RPMI 1640 medium, horse serum, NEUROBASAL^TM^ medium, B-27 supplement, Dulbecco’s Modified Eagle’s Medium (DMEM)-F12 (1:1) were supplied by Life Technologies (Grand Island, NY, USA). Trypsin was obtained from Amresco (Solon, OH, USA). The annexin V-fluorescein isothiocyanate/propidium iodide (FITC/PI) apoptosis detection kit and annexin V-FITC fluorescence microscopy kit were purchased from BD Biosciences (San Diego, CA, USA). The MMP assay kit with JC-1 (5,5′,6,6′-tetra-chloro-1,1′,3,3′-tetraethylbenzimidazol-carbocyanine iodide), the cell mitochondria isolation kit, and the bicinchoninic acid (BCA) protein assay kit were obtained from the Beyotime Institute of Biotechnology (Shanghai, China). Z-IETD-FMK and antibodies against Fas, FasL and cleaved caspase-8 were obtained from Abcam (Cambridge, MA, USA). Anti-FasL mAb (clone MFL3) was from BioLegend (San Diego, CA, USA). Antibodies against tBID and BID were purchased from Novus Biologicals (Littleton, CO USA). Antibodies against cleaved caspase-9/3, cleaved PARP, Bcl-2, Bax, cytochrome c, cyclooxygenase(COX)-IV, β-actin and horseradish peroxidase (HRP)-conjugated goat anti-rabbit immunoglobulin G (IgG) were obtained from Cell Signaling Technology (Boston, MA, USA). Fetal calf serum (FCS) and the enhanced chemiluminescence solution were purchased from Thermo Fisher Scientific (Waltham, MA USA). All other chemicals and reagents were of analytical grade.

### Cell isolation and culture

Fetal Sprague-Dawley rats at 18–19 d of gestation were obtained from the Comparative Center of Yangzhou University (Yangzhou, China). All experimental procedures were carried out in accordance with the recommendations in the Guide for the Care and Use of Laboratory Animals of the National Research Council and were approved by the Animal Care and Use Committee of Yangzhou University (approval ID: SYXK (Su) 2012–0029).

Primary cortical neurons were isolated from Sprage-Dawley rats at 18–19 d of gestation and cultured as described^18,41^. Primary neurons were used for experiments after 6 d of culture. The rat pheochromocytoma cell line, PC12 was purchased from the Type Culture Collection of the Chinese Academy of Science (Shanghai, China) and cultured in RPMI-1640 medium supplemented with 5% FCS and 10% horse serum, 1 mM L-glutamine, 100 U/mL penicillin, and 100 μg/mL streptomycin. Cells were cultured at 37 °C in 5% CO_2_.

### Hoechst 33258 Staining

After pretreated with/without 40 µM Z-IETD-FMK, a specific inhibitor of caspase-8, for 30 min and treatment with/without 10 µM Cd for 24 h, apoptotic morphological changes of primary neurons and PC12 cells were assessed by Hoechst 33258 staining as previously described^42^.

### Cell morphology detection by fluorescence microscopy analysis of annexin V-FITC staining

Primary neurons and PC12 cells were cultured at a density of 1 × 10^6^ cells/well in 6-well plates. After preincubation with/without Z-IETD-FMK (40 µM) for 30 min, cells were treated with/without 10 µM Cd for 24 h. Next, an annexin V-FITC fluorescence microscopy kit was used to assess apoptosis according to the manufacturer’s instructions. Then, the culture plates were observed under a DM13000 B inverted phase microscope (Leica, Wetzlar, Germany) using FITC and bright field filters. The cells were counted, and the number of annexin V-FITC-positive cells was expressed as a percentage of the total number of cells.

### Apoptosis detection by flow cytometry

PC12 cells were cultured in 6-well plates. Following preincubation with/without Z-IETD-FMK (40 µM) or anti-FasL (10 mg/L) for 30 min, cells were treated with/without 10 µM Cd for 24 h. After treatment, the annexin V-FITC/PI apoptosis detection kit was used to measure apoptosis according to the manufacturer’s instructions. Cells were analyzed by flow cytometry (BD Bioscience, USA). The percentage of apoptosis was calculated from early apoptosis (annexin V^+^/PI^−^, Q4) and late apoptosis (annexin V^+^/PI^+^, Q2).

### MMP (JC-1) assay

After preincubation with/without Z-IETD-FMK (40 µM) for 30 min and treatment with/without 10 µM Cd for 24 h, primary neurons and PC12 cells were collected and changes in MMP were measured using the fluorescent probe JC-1. Normally, JC-1 forms J-aggregates that emit red fluorescence in mitochondria with a higher MMP. As MMP decreases, J-aggregates shift to the monomeric form, which emits green fluorescence. Thus, a decreased ratio of red/green fluorescence (the ratio of JC-1 aggregate/monomer) suggests a decrease in MMP. Briefly, harvested cells were incubated with JC-1 in the dark for 30 min at 37 °C. Next, the cells were washed twice with JC-1 buffer solution before being imaged under a fluorescence microscope. As a positive control, cells were treated with CCCP, which significant decreases MMP.

### Cell fraction preparation

Following preincubation with/without Z-IETD-FMK (40 µM) for 30 min, primary neurons and PC12 cells were treated with Cd (0, 5, 10, or 20 µM) for 24 h, 10 µM Cd for 0, 6, 12, or 24 h. Next, cells were harvested, and cytosolic and mitochondrial fractions were prepared using the cell mitochondria isolation kit according to the manufacturer’s instructions.

### Western blot analysis

Western blot was performed as described previously^18^. After cell fraction preparation and protein quantification with a BCA protein assay kit, equal amounts of protein were separated by sodium dodecyl sulfate-polyacrylamide gel electrophoresis (SDS-PAGE) and transferred to polyvinylidene difluoride (PVDF) membranes. After blocking in 5% nonfat milk for 2 h at room temperature, the membranes were incubated overnight at 4 °C with primary antibodies against Fas, FasL, FADD, cleaved caspase-8, cleaved casepase-9/3, cleaved PARP, tBID, BID, Bcl-2, Bax, cytochrome c, COX-IV, or β-actin (1:1000 dilution), followed by incubation with the appropriate HRP-conjugated secondary antibodies (1:5000 dilution). Protein detection was performed by enhanced chemiluminescence reagent. Protein levels were determined by the Image Lab^TM^ software (Bio-Rad, Hercules, CA, USA). The density of each band was normalized to its respective loading control (β-actin or COX- IV).

### Statistical analysis

Values are presented as mean ± standard deviation (SD) from at least three independent experiments with different batches of cells. Significance was calculated by one-way ANOVA using SPSS software. The results were considered significant at *P* < 0.05 and highly significant at *P* < 0.01.

## References

[1] Wang B, Du Y (2013). Cadmium and its neurotoxic effects. Oxidative medicine and cellular longevity.

[2] Xu S (2016). Melatonin prevents abnormal mitochondrial dynamics resulting from the neurotoxicity of cadmium by blocking calcium-dependent translocation of Drp1 to the mitochondria. Journal of pineal research.

[3] Lukawski K, Nieradko B, Sieklucka-Dziuba M (2005). Effects of cadmium on memory processes in mice exposed to transient cerebral oligemia. Neurotoxicology and teratology.

[4] Nishimura Y (2006). Increase in intracellular Cd(2+) concentration of rat cerebellar granule neurons incubated with cadmium chloride: cadmium cytotoxicity under external Ca(2+)-free condition. Toxicology in vitro: an international journal published in association with BIBRA.

[5] Jiang LF, Yao TM, Zhu ZL, Wang C, Ji LN (2007). Impacts of Cd(II) on the conformation and self-aggregation of Alzheimer’s tau fragment corresponding to the third repeat of microtubule-binding domain. Biochimica et biophysica acta.

[6] Lopez E, Arce C, Oset-Gasque MJ, Canadas S, Gonzalez MP (2006). Cadmium induces reactive oxygen species generation and lipid peroxidation in cortical neurons in culture. Free radical biology & medicine.

[7] Chen L (2011). Cadmium induction of reactive oxygen species activates the mTOR pathway, leading to neuronal cell death. Free radical biology & medicine.

[8] Hossain S, Liu HN, Nguyen M, Shore G, Almazan G (2009). Cadmium exposure induces mitochondria-dependent apoptosis in oligodendrocytes. Neurotoxicology.

[9] Zhang R (2017). Celastrol Attenuates Cadmium-Induced Neuronal Apoptosis via Inhibiting Ca2+-CaMKII-Dependent Akt/mTOR Pathway. Journal of cellular physiology.

[10] Elmore S (2007). Apoptosis: a review of programmed cell death. Toxicologic pathology.

[11] Kuwana T, Newmeyer DD (2003). Bcl-2-family proteins and the role of mitochondria in apoptosis. Current opinion in cell biology.

[12] Thorburn A (2004). Death receptor-induced cell killing. Cellular signalling.

[13] Kischkel FC (1995). Cytotoxicity-dependent APO-1 (Fas/CD95)-associated proteins form a death-inducing signaling complex (DISC) with the receptor. The EMBO journal.

[14] Fulda S (2001). Cell type specific involvement of death receptor and mitochondrial pathways in drug-induced apoptosis. Oncogene.

[15] Jost PJ (2009). XIAP discriminates between type I and type II FAS-induced apoptosis. Nature.

[16] Scaffidi C (1998). Two CD95 (APO-1/Fas) signaling pathways. The EMBO journal.

[17] Nagata S (1999). Fas ligand-induced apoptosis. Annual review of genetics.

[18] Yuan Y (2013). Cadmium-induced apoptosis in primary rat cerebral cortical neurons culture is mediated by a calcium signaling pathway. PloS one.

[19] Jiang C (2014). Cadmium induces PC12 cells apoptosis via an extracellular signal-regulated kinase and c-Jun N-terminal kinase-mediated mitochondrial apoptotic pathway. Biological trace element research.

[20] Lopez E, Figueroa S, Oset-Gasque MJ, Gonzalez MP (2003). Apoptosis and necrosis: two distinct events induced by cadmium in cortical neurons in culture. British journal of pharmacology.

[21] Pradelli LA, Beneteau M, Ricci JE (2010). Mitochondrial control of caspase-dependent and -independent cell death. Cellular and molecular life sciences: CMLS.

[22] Martinou JC, Youle RJ (2011). Mitochondria in apoptosis: Bcl-2 family members and mitochondrial dynamics. Developmental cell.

[23] Liu W (2014). Calcium-calmodulin signaling elicits mitochondrial dysfunction and the release of cytochrome c during cadmium-induced apoptosis in primary osteoblasts. Toxicology letters.

[24] Yuan Y (2015). The role of mitogen-activated protein kinase in cadmium-induced primary rat cerebral cortical neurons apoptosis via a mitochondrial apoptotic pathway. Journal of trace elements in medicine and biology: organ of the Society for Minerals and Trace Elements.

[25] Wang Z (2010). Protective effect of BMP-7 against aristolochic acid-induced renal tubular epithelial cell injury. Toxicology letters.

[26] Al-Assaf AH (2013). Mechanism of cadmium induced apoptosis in human peripheral blood lymphocytes: the role ofp53, Fas and Caspase-3. Environmental toxicology and pharmacology.

[27] Pal S, Pal PB, Das J, Sil PC (2011). Involvement of both intrinsic and extrinsic pathways in hepatoprotection of arjunolic acid against cadmium induced acute damage *in vitro*. Toxicology.

[28] Oh SH, Choi JE, Lim SC (2006). Protection of betulin against cadmium-induced apoptosis in hepatoma cells. Toxicology.

[29] Liu G (2017). Beclin-1-mediated Autophagy Protects Against Cadmium-activated Apoptosis via the Fas/FasL Pathway in Primary Rat Proximal Tubular CellCulture. Scientific reports.

[30] Shetty S (2002). Tumor necrosis factor-related apoptosis inducing ligand (TRAIL) up-regulates death receptor 5 (DR5) mediated by NFkappaB activation in epithelial derived cell lines. Apoptosis.

[31] Tsuruya K (2003). Antioxidant ameliorates cisplatin-induced renal tubular cell death through inhibition of death receptor-mediated pathways. American Journal of Physiology-renal Physiology.

[32] Kantari C, Walczak H (2011). Caspase-8 and bid: caught in the act between death receptors and mitochondria. Biochimica et biophysica acta.

[33] Yin XM (1999). Bid-deficient mice are resistant to Fas-induced hepatocellular apoptosis. Nature.

[34] Li H, Zhu H, Xu CJ, Yuan J (1998). Cleavage of BID by caspase 8 mediates the mitochondrial damage in the Fas pathway of apoptosis. Cell.

[35] Akazawa Y, Gores GJ (2007). Death receptor-mediated liver injury. Seminars in liver disease.

[36] Yin XM, Ding WX (2003). Death receptor activation-induced hepatocyte apoptosis and liver injury. Current molecular medicine.

[37] Hengartner MO (2000). The biochemistry of apoptosis. Nature.

[38] Billen LP, Shamas-Din A, Andrews DW (2008). Bid: a Bax-like BH3 protein. Oncogene.

[39] Kaufmann T, Strasser A, Jost PJ (2012). Fas death receptor signalling: roles of Bid and XIAP. Cell death and differentiation.

[40] Qi F (2012). Induction of apoptosis by cinobufacini preparation through mitochondria- and Fas-mediated caspase-dependent pathways in human hepatocellular carcinoma cells. Food and chemical toxicology: an international journal published for the British Industrial Biological Research Association.

[41] Yuan Y (2012). Oxidative stress and apoptotic changes of rat cerebral cortical neurons exposed to cadmium *in vitro*. Biomed Environ Sci.

[42] Zhang Q (2014). IPS-1 plays a dual function to directly induce apoptosis in murine melanoma cells by inactivated Sendai virus. International journal of cancer.

